# Plastic bronchitis associated with respiratory syncytial virus infection: a case report

**DOI:** 10.1186/s12887-023-04351-0

**Published:** 2023-10-17

**Authors:** Wei Wang, Lei Zhang, Wei-Ke Ma, Yan-Xia He, Wen-Jian Wang, Guo-Yun Su, Jie-Hua Chen

**Affiliations:** Shenzhen Children’s Hospital, No. 7019, Yitian Road, Futian District, Shenzhen, 518026 Guangdong China

**Keywords:** Respiratory syncytial virus, Plastic bronchitis, Bronchoscopy, Child, Extracorporeal membrane oxygenation

## Abstract

**Background:**

The etiology of Plastic bronchitis (PB) is unknown. The incidence of pulmonary infection associated with PB has increased year by year, but respiratory syncytial virus (RSV) as a pathogen causes PB has rarely been reported.

**Case presentation:**

A 2-year-old immunocompromised girl was admitted to the hospital with cough, fever for 5 days, and aggravated with shortness of breath for 1 day. With mechanical ventilation, her respiratory failure was not relieved, and subcutaneous emphysema and mediastinal pneumatosis appeared. Extracorporeal membrane oxygenation (ECMO) was administrated, but the tidal volume was low. Therefore, a bronchoscopy was performed, by which plastic secretions were found and removed. Pathology of the plastic secretions confirmed the diagnosis of type I PB. RSV was the only positive pathogen in the alveolar lavage fluid by the next-generation sequencing test. After the bronchoscopic procedure, her dyspnea improved. The patient was discharged with a high-flow nasal cannula, with a pulse oxygen saturation above 95%. Half a year after discharge, she developed sequelae of bronchitis obliterans.

**Conclusion:**

RSV could be an etiology of PB, especially in an immunocompromised child. In a patient with pulmonary infection, if hypoxemia is presented and unresponded to mechanical ventilation, even ECMO, PB should be considered, and bronchoscopy should be performed as soon as possible to confirm the diagnosis and to treat.

## Background

Plastic bronchitis (PB) is a disease with a rapid onset and progression [1]. It is due to an endogenous foreign body in the airway which causes extensive bronchial blockage, resulting in pulmonary ventilation dysfunction, respiratory insufficient, and life-threatening respiratory failure. Postoperative complications and lymphatic circulation abnormalities of congenital heart diseases are common etiologies in PB in Western countries, such as the procedure of repairing congenital heart diseases, especially the Fontan procedure [2], transposition of great arteries [3], and tetralogy of Fallen [4]. Some unexplained PB cases may be caused by pulmonary lymphatic circulation abnormalities, including primary lymphangiopathies, such as lymphangiectasia and lymphangiomatosis [5]. It is rarely related to immune-mediated inflammatory reactions [6]. Pulmonary infections are common causes of PB, especially in China. Adenovirus [7, 8], influenza A virus, influenza B virus [9], *Mycoplasma pneumonia* [10], as well as bocavirus infection [11] have been reported to associate with PB in previous literature. However, to the best of our knowledge, respiratory syncytial virus (RSV) has not been reported as a pathogen in PB. Here we report a case of an immunocompromised child with RSV infection who developed fatal PB, to highlight the clinical features, diagnosis, and treatment of this rare disease.

## Case presentation

A 2-year-old female patient was admitted to Shenzhen Children’s Hospital in July 2021 because of a cough, fever for 5 days, and worsening shortness of breath for 1 day. Five days before admission, the child presented with paroxysmal productive cough, cyanosis, and fever. The highest temperature was 40.0 ℃. Four days later, the child had a worse cough and significant shortness of breath, then she was admitted to the hospital with presumptive pneumonia.

She was previously diagnosed with acute myeloid leukemia (M5, CR1) and was in the induction phase of chemotherapy, The chemotherapy regimen was cytarabine (Ara-c) 100 mg/m2 d1-7, etoposide (VP-16) 150 mg/m2 d3, cladribine (Cla) 5 mg/m2 d1-5, and granulocyte colony-stimulating factor (G-CSF) 200 µg/m2 d1-7. The patient had no history of eczema or wheezing. There was no special birth history, personal history, or family history.

The physical examination on admission was as follows: temperature 38.9 °C, heart rate 138 beats/min, respiratory rate 50 beats/min, blood pressure 104/60 mmHg, weight 12 kg, and 94% arterial oxygen saturation (with supplemental oxygen concentration 65%). The patient had poor mental status, dysphoria, shortness of breath, cyanosis, nasal flaring, retraction, wheezing, and rales on auscultation. Examinations on the heart, abdomen, and nervous system were unremarkable. The capillary refill time was 2 s.

**Fig. 1:**
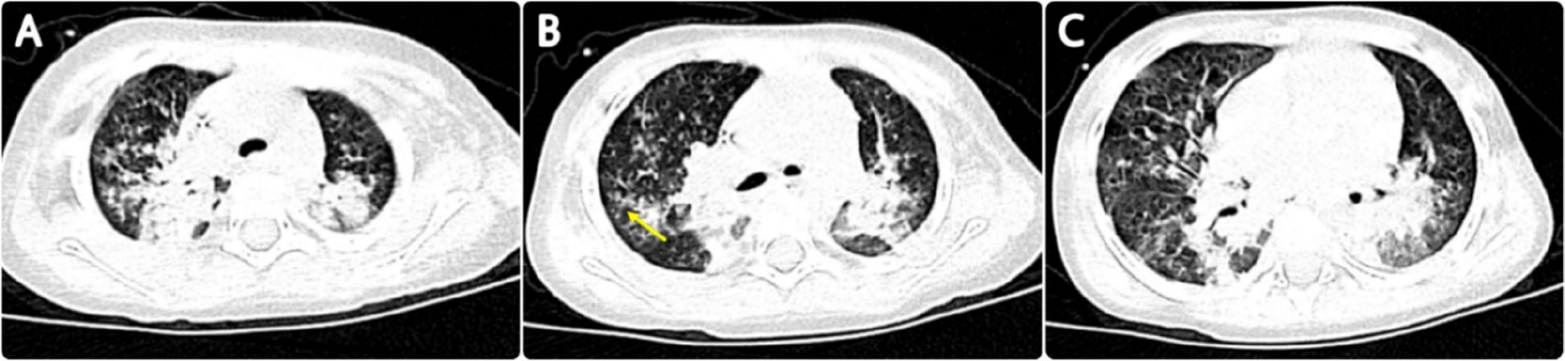
Chest CT on admission: It shows consolidation of both lungs with segmental atelectasis, centrilobular nodules, and a sign of tree-in-bud (yellow arrow)

Laboratory tests were as follows: The white blood cell counts 0.83*10^9^/L, neutrophils 0.21*10^9^/L, lymphocytes 0.62*10^9^/L, hemoglobin 105 g/L, platelet 50*10^9^/L, hypersensitive C-reactive protein 22.71 mg/L; procalcitonin 2.95 ng/ml; blood gas analysis: pH 7.403, carbon dioxide partial pressure 43.5 mmHg, oxygen partial pressure 87.4 mmHg, and standard bicarbonate 26.3 mmol/L. The standard residual base was 2.2 mmol/L. The liver and kidney function, creatinase, Brain Natriuretic Peptide, electrolyte, and coagulation function were normal; The blood culture and sputum culture were negative. The throat swab and alveolar lavage fluid PCR for respiratory pathogens (including metapneumovirus, influenza B virus, influenza A virus H3N2, Chlamydia, *Mycoplasma pneumonia*, bocavirus, coronavirus, respiratory syncytial virus (RSV), influenza A virus H1N1, adenovirus, rhinovirus, parainfluenza virus) indicated positive only for RSV only. A Chest CT (Fig. 1) suggested consolidation of both lungs with segmental atelectasis.

**Fig. 2:**
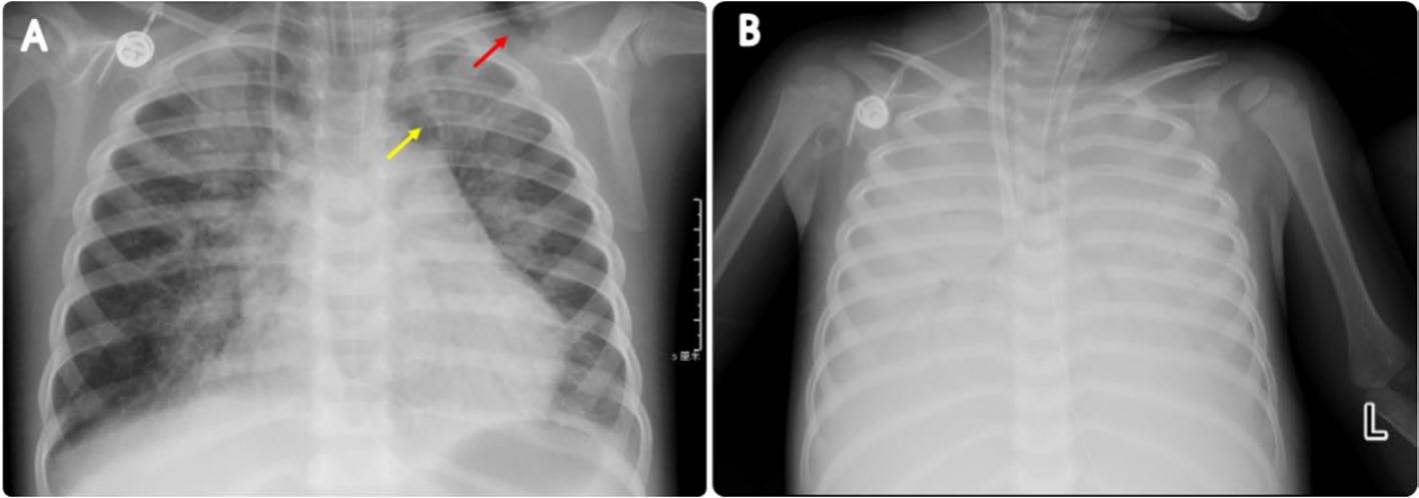
Chest X-ray during hospitalization. It shows consolidation and segmental atelectasis of both lungs (**A**), subcutaneous emphysema (red arrow), and a small amount of pneumatosis of the mediastinum (yellow arrow); It shows diffuse consolidation of both lungs (**B**), with air bronchogram sign, indicating “white lung”

**Fig. 3:**
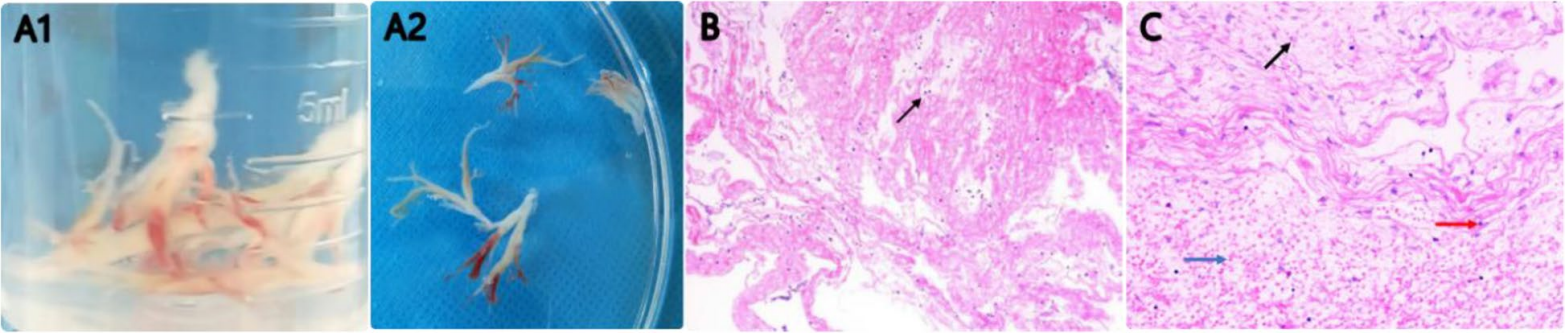
**A1-A2** Plastic secretion; **B** Low-magnification pathological image. It shows abundant fibrin and inflammatory cells (as shown by black arrows) (HE staining, ×100) (**C**) High-magnification pathological image. It shows visible neutrophils (black arrows) lymphocytes (red arrows) red blood cells (blue arrows) (HE staining, ×400)

Treatment and follow-up: During hospitalization, the child was in the induction phase of M5 chemotherapy with neutropenia and fever. Therefore, the intravenous meropenem (20 mg/kg q8 h) was given, with high-flow nasal catheter oxygen (HFNC), nebulization (with budesonide 2 ml ipratropium 2 ml salbutamol aerosol 1.25 ml once/8 h), rehydration and antipyretic. On the 6th day of hospitalization, the difficulty breathing, and retraction were worsening, and the oxygen saturation was 88% on HFNC, then she was transferred to the Pediatric Intensive Care Unit (PICU). By intubation and mechanical ventilation, the oxygen saturation was still lower than 90%, and mechanical ventilation-related lung injuries such as subcutaneous emphysema and mediastinal pneumatosis occurred (Fig. 2A). On the 9th day of hospitalization, extracorporeal membrane oxygenation (ECMO) was given, by which the tidal volume was still low, and manual lung recruitment was ineffective. Repeated chest radiographs indicated signs of “white lung” (Fig. 2B). Airway obstruction was considered, the bronchoscopy was performed on the 10th, 13th, 15th, and 19th days of ECMO operation, respectively, and many plastic plugs were aspirated during the first two procedures (Fig. 3A). After that, the tidal volume increased. The alveolar lavage fluid was sent for a high-throughput etiology test by next-generation sequencing, which was only positive for RSV. The pathology of the plastic plugs indicated fibrinous secretions, as well as red blood cells, lymphocytes, and neutrophils (Fig. 3B C). The ECMO was withdrawn 35 days later. At discharge, she was on HFNC (FiO2 34%, flow 13 L/min) with, an oxygen saturation maintained above 95%.

**Fig. 4:**
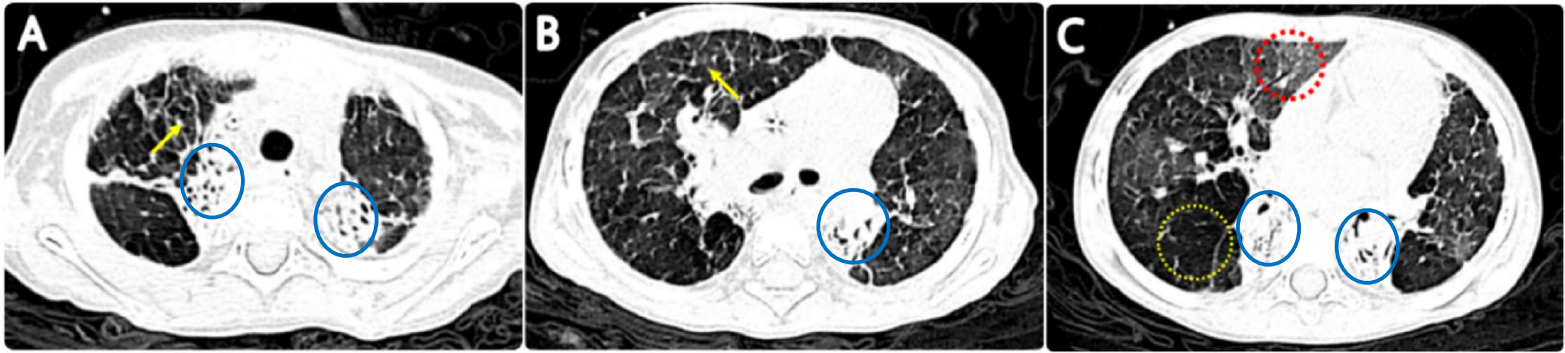
A follow-up CT scan 6 months after discharge. It shows a mosaic attenuation pattern, (hyperinflation (yellow circle) and ground glass opacity (red circle)), atelectasis (blue circle), and interlobular septal thickening (yellow arrow) in both lungs

**Fig. 5:**
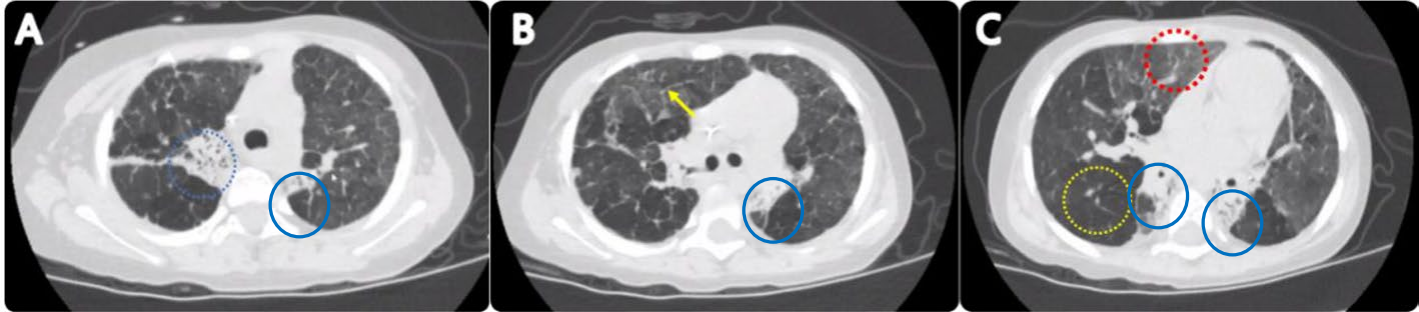
A follow-up CT scan 19 months after discharge. It still shows mosaic attenuation pattern, (hyperinflation (yellow circle) and ground glass opacity (red circle)), atelectasis (blue circle), and interlobular septal thickening (yellow arrow) in both lungs

After discharge, the child had a cough, exercise intolerance, and persistent moist rales on lung auscultation. A chest CT scan performed six months post-discharge (February 2022) revealed uneven inflation, hyperinflation, ground-glass opacity, atelectasis, and interlobular septal thickening in both lungs (Fig. 4). Consequently, bronchiolitis obliterans (BO) was diagnosed. oral montelukast sodium and low-dose azithromycin, as well as budesonide nebulization, were given. A follow-up CT scan performed 19 months post-discharge (March 2023)still indicated a mosaic attenuation pattern, atelectasis, and interlobular septal thickening (Fig. 5). In October 2022, oral pirfenidone was initiated for the treatment of pulmonary fibrosis. Currently, during more than two years of monitoring, no exacerbation of BO has been observed.

## Discussion and conclusions

PB is a critical condition that can lead to severe cardiopulmonary failure. It can affect individuals of any age but is more prevalent in children. Currently, there are no specific diagnostic criteria for PB, which relies on a combination of clinical manifestations, chest imaging, and bronchoscopic findings. Clinical manifestations and chest imaging results can vary significantly. It may be presented with cough, fever, respiratory distress, and severe systemic hypoxia. Chest imaging findings primarily depend on the extent of airway obstruction. PB can be diagnosed either when the patient coughs up bronchial casts or through the removal of a gelatinous foreign body resembling the bronchial tree via bronchoscopy.

This young child presented with an acute onset of cough, fever, and progressive dyspnea as the primary symptoms. She had a pre-existing acute myeloid leukemia M5 condition, which progressed rapidly and severely. The child’s ventilator parameters were high, and arterial oxygen saturation was challenging to maintain above 80% even supplemented with pure oxygen. ECMO procedures were performed when the patient’s oxygenation index (OI) exceeded 40, and air leakage manifestations occurred. During ECMO, bronchoscopy was performed multiple times, and early lavage removed abundant bronchial plastic secretions, and then the child’s dyspnea significantly improved. RSV infection was confirmed through a pharyngeal swab and alveolar lavage fluid. Moreover, high-throughput pathogen analysis of the alveolar lavage fluid showed RSV was the sole pathogen, which indicated acute respiratory distress syndrome (ARDS) and PB were probably associated with RSV infection. RSV is known to be clinically severe in immunocompromised patients [12, 13]. In this case, RSV-induced ARDS may be attributed to the underlying acute myeloid leukemia and compromised immunity, leading to a high viral load, prolonged detoxification, and severe lung lesions. Previous studies [14, 15] have demonstrated that RSV infection causes varying degrees of alveolar damage, hyaline membrane formation, alveolar epithelial damage, and extensive neutrophil infiltration in the alveoli, which may be associated with increased permeability due to damage to the alveolar capillary barrier. RSV infection in children can result in mucus, cellulose, and necrotic cell blockage in the bronchioles, as well as inflammatory cell infiltration in the small bronchi and alveoli, leading to the abnormal accumulation and formation of plastic secretions in the airways. Hence, RSV may be one of the etiological factors causing BO. This child had an underlying hematological neoplastic disease, and low immunity may be a contributing factor. The clinical symptoms of PB [8] primarily include cough, fever, shortness of breath, and dyspnea. Imaging features mainly consist of extensive pulmonary consolidation and/or atelectasis. In patients with respiratory distress and diffuse lesions on chest imaging, PB should be considered, especially when respiratory support is ineffective. In this case, fibrinous exudates, lymphocytes, neutrophils, and other components were found in the pathology of plastic secretions. According to Madsen (2005) [16], this child is hematologically ill, immunocompromised, and susceptible to unusual accumulation of mucus and fibrin in the airways following inflammatory insults.

ECMO is commonly employed in patients with severe respiratory diseases, as it enhances lung ventilation and oxygenation while providing time for lung injury repair. In this pediatric case of ARDS, ECMO not only ameliorated hypoxemia but potentially prevented pulmonary barotrauma resulting from elevated ventilator parameters. PB was diagnosed in this child, who exhibited low tidal volume during ECMO and abundant plastic secretions in the trachea, as observed via bronchoscopy. It is recommended that PB should be considered when advanced respiratory support fails to improve a patient’s respiratory failure. PB treatment primarily involves prompt removal of plastic secretions from the airway and optimization of ventilation. Following multiple bronchoscopic removals of plastic secretions, ventilator parameters were reduced, and tidal volume increased, which indicated effective treatment. However, postinfectious BO developed during follow-up after discharge, which may be attributed to severe lung and airway damage during the acute phase. Patients with severe pneumonia necessitating ECMO have a high incidence of postinfectious BO [17].

In conclusion, this case indicates that RSV may be a potential etiological agent for PB, particularly in immunocompromised patients. In this case, if interventions such as mechanical ventilation and ECMO fail to alleviate symptoms, the diagnosis of PB and a bronchoscopy procedure should be considered. Timely removal of the plastic secretions in the airway is crucial for treatment.

## Data Availability

All data generated or analyzed during this study are included in this published article.

## Abbreviations

PB: Plastic bronchitis
RSV: Respiratory syncytial virus
BO: Bronchiolitis obliterans
HFNC: High-flow nasal cannula
ECMO: Extracorporeal membrane oxygenation
OI: Oxygenation index
ARDS: Acute respiratory distress syndrome
PICU: Pediatric Intensive Care Unit
